# Preclinical Studies of a Rare CF-Causing Mutation in the Second Nucleotide Binding Domain (c.3700A>G) Show Robust Functional Rescue in Primary Nasal Cultures by Novel CFTR Modulators

**DOI:** 10.3390/jpm10040209

**Published:** 2020-11-05

**Authors:** Onofrio Laselva, Jacqueline McCormack, Claire Bartlett, Wan Ip, Tarini N. A. Gunawardena, Hong Ouyang, Paul D. W. Eckford, Tanja Gonska, Theo J. Moraes, Christine E. Bear

**Affiliations:** 1 Programme in Molecular Medicine, Hospital for Sick Children, Toronto, ON M5G 8X4, Canada; onofriolaselva@gmail.com (O.L.); jackie_kidd21@hotmail.com (J.M.); tarini.gunawardena@sickkids.ca (T.N.A.G.); peckford@uoguelph.ca (P.D.W.E.); 2 Department of Physiology, University of Toronto, Toronto, ON M5G 8X4, Canada; 3 Programme in Translational Medicine, Hospital for Sick Children, Toronto, ON M5G 8X4, Canada; claire.bartlett@sickkids.ca (C.B.); wan.ip@sickkids.ca (W.I.); hong.ouyang@sickkids.ca (H.O.); tanja.gonska@sickkids.ca (T.G.); theo.moraes@sickkids.ca (T.J.M.); 4 Department of Paediatrics, University of Toronto, Toronto, ON M5G 8X4, Canada; 5 Department of Biochemistry, University of Toronto, Toronto, ON M5G 8X4, Canada

**Keywords:** cystic fibrosis, CFTR, rare mutations, c.3700A>G, TRIKAFTA, novel modulators, personalized medicine, patient-derived nasal epithelial culture

## Abstract

The combination therapies ORKAMBI^TM^ and TRIKAFTA^TM^ are approved for people who have the F508del mutation on at least one allele. In this study we examine the effects of potentiator and corrector combinations on the rare mutation c.3700A>G. This mutation produces a cryptic splice site that deletes six amino acids in NBD2 (I1234-R1239del). Like F508del it causes protein misprocessing and reduced chloride channel function. We show that a novel cystic fibrosis transmembrane conductance regulator CFTR modulator triple combination (AC1, corrector, AC2-2, co-potentiator and AP2, potentiator), rescued I1234-R1239del-CFTR activity to WT-CFTR level in HEK293 cells. Moreover, we show that although the response to ORKAMBI was modest in nasal epithelial cells from two individuals homozygous for I1234-R1239del-CFTR, a substantial functional rescue was achieved with the novel triple combination. Interestingly, while both the novel CFTR triple combination and TRIKAFTA^TM^ treatment showed functional rescue in gene-edited I1234-R1239del-CFTR-expressing HBE cells and in nasal cells from two CF patients heterozygous for I1234-R1239del/W1282X, nasal cells homozygous for I1234-R1239del-CFTR showed no significant response to the TRIKAFTA^TM^ combination. These data suggest a potential benefit of CFTR modulators on the functional rescue of I1234-R1239del -CFTR, which arises from the rare CF-causing mutation c.3700A>G, and highlight that patient tissues are crucial to our full understanding of functional rescue in rare CFTR mutations.

## 1. Introduction

The Cystic Fibrosis transmembrane conductance regulator, CFTR, mediates PKA-regulated, ATP-dependent chloride and bicarbonate flux across apical membranes of polarized epithelial cells of organs, such as the lungs, pancreas and intestine [1,2,3]. Cystic fibrosis (CF), which affects approximately 80,000 people worldwide, is caused by mutations in the *CFTR* gene which result in defects in protein quantity, biosynthetic processing, stability, regulation and/or channel gating. While >2000 mutations have been identified in the *CFTR* gene, caused mainly by missense, frameshift, splicing or nonsense mutations [4], only about three hundred have so far been characterized as disease-causing [5]. A highly effective small molecule modulator therapy has been FDA-approved for the major mutant, F508del-CFTR, present in >90% of North American patients in heterozygous or homozygous form (TRIKAFTA^TM^) [6], and for about 38 rare mutations primarily of the gating type present in approximately 10% of North American patients (KALYDECO^TM^; ivacaftor) [6,7,8,9,10]. However, not all mutations can be treated by these two therapies, and it is not yet clear if these therapies are the most effective possible even for F508del and gating mutations.

A significant number of CF patients worldwide lack F508del or KALYDECO^TM^-approved mutations on both alleles and remain without CFTR modulator therapy. While many of these mutations may be considered rare, regional or founder population effects may cause certain mutations to be more prevalent within groups or areas. Examples include the W1282X mutation in Jewish populations, M1101K among Hutterite communities, and c.3700G>A, a mutation common in individuals with ancestry from Bedouin tribes and the second most common CF mutation in the Middle East [11,12,13].

The *CFTR* variant c.3700A>G has been predicted to create a missense mutation (p.Ile1234Val). As we demonstrated, I1234V-CFTR caused no change in the CFTR protein folding, processing and function when expressed in an overexpression system [14]. Detailed analysis of *CFTR* gene and *CFTR* mRNA obtained from nasal cultures of a CF patient homozygous for c.3700A>G, showed that this mutation activates a cryptic donor splice site (with no wild-type (WT) transcript), resulting in deletion of six amino acids (I1234-R1239del) in NBD2, which like the F508del mutation, was shown to cause a primary defect in folding and processing [14]. However, treatment of primary nasal epithelial cultures from two individuals homozygous for the c.3700A>G mutation with the first generation F508del modulator therapy, ORKAMBI^TM^ (lumacaftor + ivacaftor), failed to rescue functional expression of this mutant to a level anticipated to be therapeutically relevant. In a subsequent study, it was shown that “amplifier” therapy meant to stabilize CFTR mRNA, in combination with the components of ORKAMBI^TM^ in in vitro studies of patient nasal epithelial cultures increased the levels of immature CFTR protein and significantly increased the ORKAMBI^TM^ response [15]. This suggests that this mutation may be effectively treated clinically by the right combination of CFTR modulator molecules. A recent study by Phuan and colleagues [16], showed I1234-R1239del-CFTR functional rescue by TRIKAFTA^TM^ in I1234-R1239del-CFTR expressing Fisher Rat Thyroid (FRT) cells, gene-edited Human Bronchial Epithelial (HBE) cells expressing I1234-R1239del-CFTR and in primary nasal cultures from sibling CF donors bearing I1234-R1239del/W1282X mutations. Interestingly, they found that nasal cultures from a donor homozygous for c.3700A>G did not show CFTR functional rescue by TRIKAFTA^TM^. They also evaluated the effects of TRIKAFTA^TM^ treatment on two individuals homozygous for the c.3700A>G mutation. This study showed some improvements in both patients, with one showing a decrease in sweat chloride and symptomatic improvement, and the other showing a small improvement in lung function. Results from further follow up studies on these and additional individuals are awaited.

Small molecule CFTR therapies are in development from a variety of companies, including Vertex, Abbvie, Proteostasis and others. While it would be prohibitively expensive and difficult to develop a separate modulator therapy for every mutation, theratyping of different mutations in overexpression systems and follow-up with patient tissues may prove an effective pipeline to allow repurposing of modulators for all CF mutations where they may be effective. We and other groups have examined modulators, approved or in various stages of clinical trials, in vitro on patient tissues possessing various rare *CFTR* mutations outside of their current indications [15,16,17,18,19,20]. Abbvie has developed a combination of CFTR correctors and potentiators, some of which are currently in clinical trials for CF patients bearing the F508del mutation. We have earlier studied related compounds (AC1, AC2-1 and AC2-2) and have shown that they can significantly augment W1282X-CFTR function in patient nasal tissues (in the presence of an inhibitor of nonsense-mediated decay), and that they may be effective in other mutations, including the processing mutations M1101K, G85E and N1303K [17,21]. Moreover, we demonstrated that one compound, AC2-2, exhibits dual activities as a corrector and a potentiator for M1101K and G85E, but only functions as a potentiator for N1303K [21].

In this study we examine the effects of the novel Abbvie modulators and TRIKAFTA^TM^ on the rare c.3700A>G mutation in Human Embryonic Kidney (HEK)-293 cells and 16HBE cells. We also tested the small molecule combinations in patient derived nasal epithelial cultures from two patients homozygous for I1234-R1239del and two heterozygous patients I1234-R1239del/W1282X. We found that the specific modulator combination (AC1 + AC2-2 + AP2) rescued functional expression of I1234-R1239del-CFTR to normal function in patient-derived nasal epithelial cells.

## 2. Materials and Methods

### 2.1. Cell Culture and Transfection

Human embryonic kidney (HEK) 293 GripTite™ cells (HEK293) (a gift from Dr. Daniela Rotin, Hospital for Sick Children, Toronto, ON, Canada) were maintained as previously described [22,23].

I1234_R1239del/W1282X nasal epithelial cells were obtained through Dr. Carlos Milla at the CF Center at Stanford University, and I1234_R1239del homozygous nasal cells were obtained from family members at The Hospital for Sick Children (after obtaining informed consent). The subsequent nasal epithelial cell culture was performed as previously described [24,25,26]. Cells were seeded on collagen coated transwell inserts and once confluent, the cells were cultured for 14 days at an air liquid interface (ALI) with basal differentiation media (PneumaCult^TM^ ALI, StemCell Tech., Vancouver, BC, Canada).

### 2.2. Compound Description

The small molecule modulators of CFTR used in this study were: VX-770, VX-661 and VX-809 (Selleck Chemicals, Houston, TX, USA), VX-445 (S-enantiomer; MedChemExpress, Monmouth Junction, NJ, USA [27]), CFTR-specific inhibitor 172 (CFTRinh-172) (Cystic Fibrosis Foundation Therapeutics).

Some of the Abbvie compounds used in this study are structurally related to clinical candidates currently in clinical trials for subjects who are homozygous for F508del and were obtained by Abbvie Inc., North Chicago, IL, USA. Corrector AC1 (X281602) is described as compound 72 in a recently published review by Kym et al. [28]. Corrector AC2-1 (X281632) belongs to the ABBV/GLPG2737 series type C2 correctors belonging to the pyrazolopyridine acylsulphonamide chemical class correctors and is a close analog of compound 93 described in the review by Kym et al. [28] and covered in the patent granted to Galapagos and Abbvie (Patent WO2017060874A1; 2017). Corrector AC2-2 (X300549) belongs to the ABBV/GLPG3221 series type C2 correctors belonging to the pyrrolidine chemical class of correctors and is described as Compound 4 in an Abbvie manuscript [29] and is a close analog of ABBV/GLPG3221 [30]. Potentiator AP2 (X300529) belongs to the sulphonamide-substituted aminopyridines class of potentiators and is a close analog of GLPG2451 [31] and compound 48 described in the review by Kym et al. and covered in a patent granted to Galapagos and Abbvie (Patent WO2016193812A1, 2016).

### 2.3. CFTR Channel Function in HEK Cells

HEK293 cells were seeded in 96-well plates (black, flat bottom; Greiner) transfected with either WT- or I1234_R1239del-CFTR constructs as previously described [32,33]. The cells were treated with 0.1% Dimethyl sulfoxide (DMSO) or CFTR correctors for 24 h and Fluorometric imaging plate reader (FLIPR) buffer for 35 min at 37 °C [34]. The plate was then read in a fluorescence plate reader (excitation: 530 nm, emission: 560 nm; SpectraMax i3; Molecular Devices) at 37 °C, and after reading the baseline fluorescence for 5 min; CFTR was stimulated using forskolin (10 µM; Sigma–Aldrich, St. Louis, MO, USA) and the potentiators VX-770 (1 µM) or AP2 (1.5 µM). CFTR inhibitor (CFTRinh-172, 10 µM) was then added to inactivate CFTR. The peak changes in fluorescence to CFTR agonists were normalized relative to fluorescence immediately before agonist (forskolin) addition [35].

### 2.4. CFTR Channel Function in HBE Cells

16HBE cells, a gift from Dr. D.C. Gruenert (USF), were used for Clustered Regularly Interspaced Short Palindromic Repeats (CRISPR)/Cas9-mediated gene editing to introduce the I1234_1239del mutation as previously described [15]. I1234_R1239del-CFTR HBE cells were grown at 37 °C for five days post-confluence submerged on 96-well black, clear bottom culture plates (Costar) as previously described [15]. The HBE cells were treated with DMSO, 3 µM VX-809, 3µM VX-661 + 3 µM VX-445, 0.5 µM AC1 + 3µM AC2-1, 0.5 µM AC1 + 3µM AC2-2 for 24 h. The cells were then loaded with blue membrane potential dye dissolved in chloride-free buffer (150 mM NMDG-gluconate, 3 mM potassium gluconate, 10 mM HEPES, pH 7.30, 300 mOsm) and read in a fluorescence plate reader (SpectraMax i3; Molecular Devices) at 37 °C (excitation: 530 nm; emission: 560 nm). 10 µM forskolin was added to stimulate CFTR with 1 µM VX-770 for cells treated with VX-809 or VX-661 + VX-445 or 1.5 µM AP2 for cells treated with AC1 + AC2-1 and AC1 + AC2-2. Finally, 10 µM CFTRinh-172 was added to inactivated CFTR [36].

### 2.5. Ussing Chamber Studies of Primary Nasal Epithelial Cells

Primary nasal epithelial cultures were studied in a non-perfused Ussing chamber (Physiologic Instruments, San Diego, CA, USA) after 48 h treatment with 0.1% DMSO, 3 µM VX-809, 0.5 µM AC1 + 3 µM AC2-1, 0.5 µM AC1 + 3 µM AC2-2 or 3 µM VX-661 + 3 µM VX-445. The buffer solution (126 mM NaCl, 24 mM NaHCO_3_, 2.13 mM K_2_HPO_4_, 0.38 mM KH_2_PO_4_, 1 mM MgSO_4_, 1 mM CaCl_2_ and 10 mM glucose) was maintained at pH 7.4 and 37 °C and continuously gassed with a 5% CO_2_/95% O_2_ mixture. The transepithelial potential (Vte) was recorded in open-circuit mode and the baseline resistance (Rte) was measured following repeated, brief short-circuit current pulses (1 µA every 30 s). The results are presented as equivalent transepithelial current (Ieq), which was calculated using Ohm’s law. Following the epithelial sodium channel (ENaC) inhibition with 30 µM of Amiloride (Spectrum Chemical, Gardena, CA, USA), CFTR was stimulated with 10 µM forskolin and 1 µM VX-770 for cells treated with VX-809 or VX-661 + VX-445, or 1.5 µM for AP2 for cells treated with AC1 + AC2-2. Then, CFTR was inhibited with 5 µM CFTRInh-172 (EMD Millipore Corp., Billerica, MA, USA) [15,24].

### 2.6. Statistical Analysis

Data are represented as mean ± S.E.M. GraphPad Prism 7.0 software (San Diego, CA, USA) was used for all statistical tests. One-way ANOVA were conducted as appropriate, and *p*-values < 0.05 were considered significant. Data with multiple comparisons were assessed using Tukey’s multiple-comparison test with α = 0.05. Spearman correlation was used for correlation analysis.

## 3. Results

### 3.1. Effects of CFTR Modulators on the CFTR Function of I1234_R1239del-CFTR in HEK293 Cells

**Figure 1:**
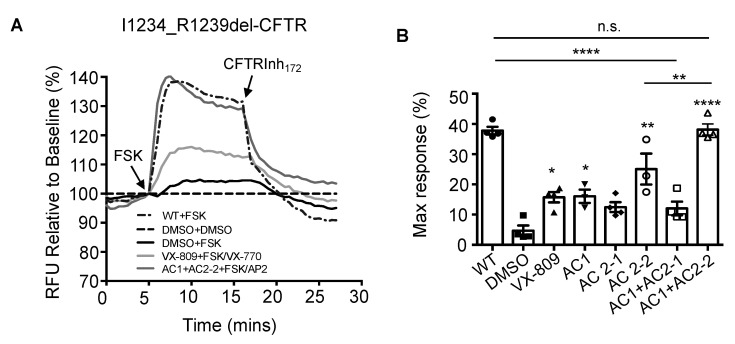
Novel triple combination of CFTR modulators completely rescue the functional expression of I1234_R1239del-CFTR in HEK293 cells. (**A**) Representative traces of I1234_R1239del-CFTR-dependent chloride efflux using the imaging plate reader membrane depolarization assay (FLIPR). HEK293 cells were pre-treated with (DMSO), 3 µM VX-809, 0.5 µM AC1 + 3 µM AC2-1, 0.5 µM AC1 + 3 µM AC2-2 for 24 h at 37 °C. Following 5 min baseline measurement, 10 µM FSK +/− 1µM VX-770 or 1.5 µM AP2 were added. After approximately 10 min incubation, CFTR inhibitor (CFTRinh-172, 10 µM) was added to deactivate CFTR, as noted by the change in the slope of the curves. (**B**) Bar graphs show the mean (±SEM) of maximal activation of CFTR after stimulation by forskolin FSK +/− Potentiators (VX-770 or AP2) (*n* = 4 biological replicates with the symbols being the mean of 4 technical replicates). (* *p* < 0.05; ** *p* < 0.01; **** *p* < 0.0001).

The CFTR mutation I1234_R1239del-CFTR was expressed in HEK293 cells and the effects of various modulators on its chloride channel function and protein processing were examined. Regulated CFTR chloride channel function was measured using the FLIPR assay as the membrane depolarization stimulated by forskolin addition in cells exposed to an outward chloride gradient. As expected for a CFTR mediated effect, the depolarization was reversed by the addition of CFTRInh-172. As previously demonstrated [15], we saw reduced forskolin-dependent channel activity of I1234_R1239del-CFTR relative to the Wt-CFTR protein (Figure 1A). Pre-treatment of the cells expressing I1234_R1239del-CFTR with the corrector compound VX-809 (lumacaftor), or the novel corrector compounds AC1, AC2-1 and AC2-2 alone all resulted in significant improvements in potentiated channel activity (Figure 1A,B). When a combination of two compounds were used the greatest potentiator effect was seen for the AC1 and AC2-2 compound combination with the AC1 and AC2-1 combination being no better than AC2-1 alone.

### 3.2. Effects of CFTR Modulators on the CFTR Channel Function in Nasal Epithelial Cells Derived from Two Patients Homozygous for the I1234_R1239del-CFTR Mutation

**Figure 2:**
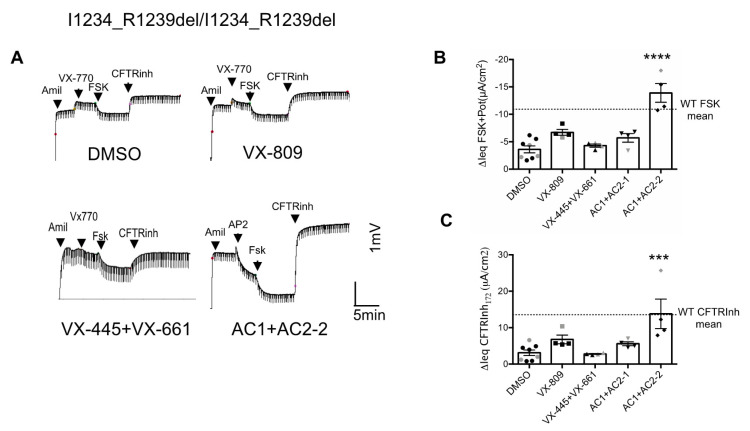
CFTR modulators rescued CFTR channel function in nasal epithelial cells derived from two patients homozygous for the I1234_R1239del-CFTR mutation. (**A**) Representative tracing show Ussing chamber measurements of transepithelial potential difference trace of I1234_R1239del-CFTR in nasal cell cultures pre-treated with DMSO, 3 µM VX-809, 3 µM VX-445 + 3 µM VX-661, 0.5 µM AC1 + 3 µM AC2-1, 0.5 µM AC1 + 3 µM AC2-2 for 48 h at 37 °C. (**B**) Bar graph showing mean peak responses (Ieq (μA/cm^2^ ± SEM) to forskolin (10 µM) and VX-770 (1 µM) or AP2 (1.5 µM) activated Ieq for nasal cultures from 2 patients after pre-treatment for 48 h with CFTR correctors. Modulators were tested in 1–4 cultures for each patient with each data from each patient showed as grey or black symbols, peak value for culture shown as dot. (**C**) Bar graphs showing the IeqCFTR inhibition (µA/cm^2^) by CFTRInh-172 (10 µM). (*** *p* < 0.001, **** *p* < 0.0001).

Next, we tested the effects of the modulators on the function of CFTR in nasal epithelial cultures from two patients homozygous for the I1234_R1239del-CFTR mutation. As for the HEK293 cells, preincubation with VX-809, the combination of AC1 and AC2-1 and the combination of AC1 and AC2-2 all resulted in improvements in CFTR channel activity. The greatest improvement was again seen for the treatment with the combination of AC1 and AC2-2, which rescued I1234_R1239del-CFTR up to ~130% of the mean forskolin response in non-CF cultures (Figure 2A,B). This could reflect the previous observation we have made that AC2-2 can act as a dual potentiator and corrector in some CFTR mutations [21]. Moreover, the combination of AC1+AC2-2 mediated a similar CFTRInh-172 response in non-CF (WT) cultures (Figure 2C).

Recently the triple compound combination, TRIKAFTA^TM^, which contains two correctors (VX-445 and VX661) and a potentiator (VX770), has been approved by the Food and Drug Administration (FDA) for the treatment of CF patients with at least one copy of the F508del mutation. Therefore, we compared the effects of TRIKAFTA^TM^ with the combination of AC1, AC2-2 and AP2 on the functional rescue of I1234_R1239del-CFTR in nasal epithelial cultures obtained from individuals homozygous for the I1234_R1239del mutation. Interestingly, in agreement with a recent studies by Phuan and colleagues [16], TRIKAFTA^TM^ did not rescue the I1234_R1239del-CFTR function in nasal epithelial cultures (Figure 2A–C) but did significantly rescue I1234_R1239del-CFTR in CRISPR/Cas9 edited human bronchial epithelial (HBE) cells (Figure S1).

### 3.3. Nasal Epithelial Cultures Derived from Patients Bearing the Heterozygous CFTR Genotype I1234_R1239del/W1282X Show a Modest CFTR Rescue by the Novel Modulator Combination and TRIKAFTA^TM^

**Figure 3:**
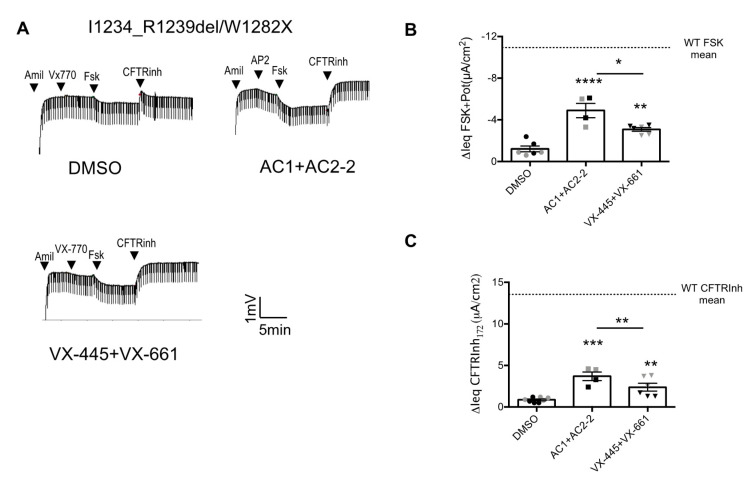
Nasal epithelial cultures derived from 2 patients bearing I1234_R1239del/W1282X mutation showed a minimal CFTR rescue by the novel triple combination. (**A**) Representative tracing show Ussing chamber measurements of transepithelial potential difference trace of I1234_R1239del/W1282X-CFTR in nasal cell cultures pre-treated with DMSO, 3 µM VX-661 + 3 µM VX-445 or 0.5 µM AC1 + 3µM AC2-2 for 48 h at 37 °C. (**B**) Bar graph showing mean peak responses Ieq (μA/cm^2^ ± SEM) to forskolin (10 µM) and VX-770 (1 µM) or AP2 (1.5 µM) activated Ieq for nasal cultures from 2 patients after pre-treatment for 48 h with CFTR correctors Modulators were tested in 2–3 cultures for each patient with each data from each patient showed as grey or black symbols; peak value for culture shown as dot. (**C**) Bar graphs showing the IeqCFTR inhibition (µA/cm^2^) by CFTRInh-172 (10 µM). (* *p* < 0.05, ** *p* < 0.01, *** *p* < 0.001, **** *p* < 0.0001).

As shown in Ussing chamber measurements in Figure 3A,B, forskolin evoked transepithelial currents in nasal epithelia from two sibling I1234_R1239del/W1282X patients were improved by treatment with AC1 and AC2-2 or with VX-445 and VX-661 versus DMSO vehicle control, with the best response seen for the combination of AC1 and AC2-2. While the novel AC1/AC2-2/AP2 CFTR modulator combination showed the best function in I1234_R1239del/W1282X-CFTR nasal cells, the improvement for these heterozygous cells was only ~50% of the mean forskolin response (Figure 3B) and ~30% of the mean CFTRInh-172 response observed in non-CF cultures (Figure 3C).

Since we previously showed that nasal epithelial cultures from CF donors homozygous for W1282X mutation lacked significant CFTR protein expression after treatment with correctors alone [17], the functional rescue by CFTR modulators is anticipated to be primarily due the c.3700A>G allele.

## 4. Discussion

We previously demonstrated that the I1234_R1239del mutation has the potential for increased functional rescue using an “amplifier” compound to augment the ORKAMBI^TM^ response in nasal epithelial cells and an HBE cell line [15]. We have also shown that AC1 stabilizes the protein fragment corresponding to MSD1, AC2-1 stabilizes MSD2 and AC2-2 stabilizes NBD2 [17]. AC2-2 displays dual activities as a corrector and potentiator for some mutations [21]. In the current studies we show the use of a strategic combination of CFTR modulators, including AC2-2, induces the functional rescue of the I1234_R1239del mutation in homozygous patient-derived nasal epithelial cells to wildtype levels. The inclusion of AC2-2 likely acts to overcome the defect caused by this NBD2 mutation, or there may be benefits to the addition of a third compound with potentiator and corrector functions (i.e., AC2-2, [21]). Similar results were obtained by Phuan et al. [16] where the addition of a co-potentiator after VX-770 further increased the I1234_R1239del-CFTR activity in FRT and HBE cells, and in nasal epithelial cultures from two donors bearing I1234_R1239/W1282X, pre-treated with VX-661 + VX-445.

Lukacs and group have demonstrated that VX-661 and VX-809 are type I correctors that stabilize the MSD2-NBD1 interface, and other correctors stabilize NBD1 (type III) or NBD2 (type II) [20,37]. VX-445 synergistically rescues F508del-CFTR folding with type I and II correctors but not with a type III corrector, suggesting that it may be a class III corrector that stabilizes NBD1 [38], which may not be as advantageous in the c.3700A>G NBD2 mutation.

Here we show that the combination of VX-770, VX-661 and VX-445 improved functional rescue of the I1234-R1239del mutant in nasal cultures from two patients heterozygous for the mutation (I1234_R1239del/W1282X) but not in nasal cultures from two patients homozygous for c.3700A>G, in agreement with the previous study by Phuan et al. [16]. This finding was somewhat surprising given the positive effect of TRIKAFTA^TM^ in the HBE cell line that was edited to harbor this mutation on both alleles. We hypothesized that this discrepancy could be due to the differential genetic factors (such as modifier genes) or environmental factors at play among the 4 participant samples and HBE cell line that could induce different residual CFTR protein levels. The participant disease status at the time of nasal brushing, or brush method differences between the two locations where the nasal samples were collected, could also be factors.

Interestingly, despite the lack of robust responses in-vitro, clinical treatment of the two patients, homozygous for c.3700A>G with TRIKAFTA^TM^ led to modest symptomatic responses [16]. In fact, in vivo TRIKAFTA treatment of two patients showed a small improvement in the lung function only in one patient [16]. This study suggests that in vitro assays on patient-specific tissues may not always predict clinical outcomes.

The G551D-CFTR mutation was the first with a highly effective modulator therapy in KALYDECO^TM^ [39]. The F508del mutation required a triple combination of two different correctors and a potentiator: TRIKAFTA^TM^ [7]. Mechanistically different correctors and potentiators, such as those described here, amplifiers that stabilize mRNA levels, such as PTI-428 [15], inhibitors of nonsense-mediated decay for mutations such as W1282X [17,40], and new compounds with unique mechanisms of action may be required in different combinations to address many of the remaining several hundred clinically relevant CFTR mutations that account for 5–10% of CF patients not eligible for KALYDECO^TM^ or TRIKAFTA^TM^.

In this work, we demonstrated the utility and value of comparing different combinations of CFTR modulators and drugs using nasal epithelial tissues from individuals bearing a rare CF-causing mutations [15,17,21,24,25]. Such studies reveal the potential for variable responses amongst individuals for existing drugs as well as the potential for emerging interventions to exert a superior effect. We propose that drug combinations should be tested on patient tissues to empirically determine the most highly effective modulator therapy for each patient.

## Supplementary Materials

The following are available online at https://www.mdpi.com/2075-4426/10/4/209/s1, Figure S1: F508del-CFTR correctors rescued CFTR function in HBE cells edited by CRISPR/Cas9 to express I1234_R1239del-CFTR.
