# Supplementary material for: Preclinical Studies of a Rare CF-Causing Mutation in the Second Nucleotide Binding Domain (c.3700A>G) Show Robust Functional Rescue in Primary Nasal Cultures by Novel CFTR Modulators
Source: J Pers Med. 2020 Nov 5;10(4):209. doi: 10.3390/jpm10040209 (PMC7712331; doi:10.3390/jpm10040209)
Supplement: Supplementary file 1 [file jpm-10-00209-s001.pdf]

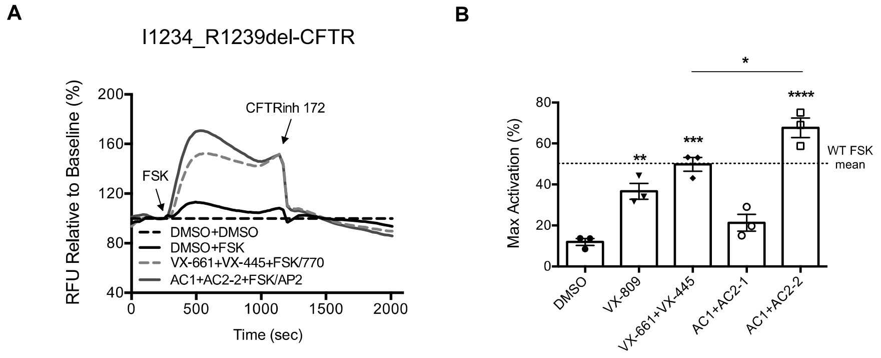

Figure S1. F508del-CFTR correctors rescued CFTR function in HBE cells edited by CRISPR/Cas9 to express I1234_R1239del-CFTR. (A) Representative traces of I1234_R1239del-CFTR-dependent chloride efflux in HBE cells using the imaging plate reader membrane depolarization assay (FLIPR). HBE cells were pre-treated with DMSO, 3μM VX-809, 3μM VX-661 + 3μM VX-445, 0.5μM AC1+ 3μM AC2-1, 0.5μM AC1+ 3μM AC2-2 for 24h at 37°C. (B) Bar graphs show the mean (±SEM) of maximal activation of CFTR after stimulation by FSK and Potentiators (1μM VX-770 or 1.5 μM AP2) (n = 4 biological replicates with the symbols being the mean of 4 technical replicates). (**$p<0.01$; ***$p<0.001$; ****$p<0.0001$)
